# Supplementary material for: Disordered-to-ordered transitions in assembly factors allow the complex II catalytic subunit to switch binding partners
Source: Nat Commun. 2024 Jan 11;15:473. doi: 10.1038/s41467-023-44563-7 (PMC10784507; doi:10.1038/s41467-023-44563-7)
Supplement: Supplementary file 1 — Supplementary Information [file 41467_2023_44563_MOESM1_ESM.pdf]

Disordered-to-ordered transitions in assembly factors allow the Complex II catalytic subunit to switch binding partners

Pankaj Sharma,[1] Elena Maklashina,[2,3] Markus Voehler,[4,5] Sona Balintova[6,7], Sarka Dvorakova[6], Michal Kraus[6], Katerina Hadrava Vanova[6,8], Zuzana Nahacka[6], Renata Zobalova,[6] Stepana Boukalova,[6] Kristyna Cunatova[9], Tomas Mracek,[9] Hans K. Ghayee,[10] Karel Pacak,[8] Jakub Rohlena,[6] Jiri Neuzil[6,7,11,12,*], Gary Cecchini[2,3,*], T. M. Iverson[1,5, 13,14,*]

Content

1. Supplementary Figures 1 – 14
2. Supplementary Tables 1 – 4

Supplementary Figures 1 – 14

Supplementary Figure 1

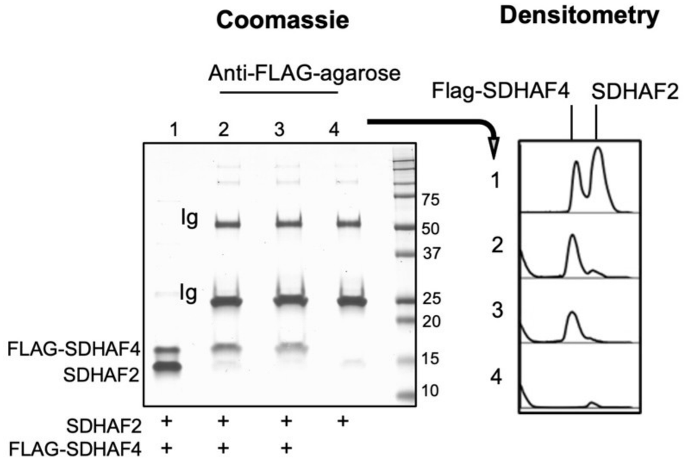

Supplementary Figure 1. SDHAF2 and SDHAF4 do not associate above the level of a negative control. The interaction between FLAG-SDHAF4 (4 µM) and SDHAF2 (8 µM) was evaluated by immunoprecipitation with Anti-FLAG-M2 Affinity gel. SDS-PAGE shows representative samples (left) and their densitometry scans (right). Lane 1 shows the SDHAF2 and FLAG-SDHAF4 input. Lanes 2 and 3 are duplicate immunoprecipitation experiments. Lane 4 is a control showing that the anti-FLAG-M2 antibody interacts with SDHAF2 at a level similar to what is observed in the pull-downs with SDHAF4 present. This is consistent with SDHAF2 interacting non-specifically with the resin. As SDHAF4 does not pull-down SDHAF4 above the level of a negative control, the result suggests that SDHAF2 and SDHAF4 do not interact in the absence of SDHA. The SDS-PAGE gel is shown the representative example from three independent experiments. Source data are provided as a Source Data file.

Supplementary Figure 2

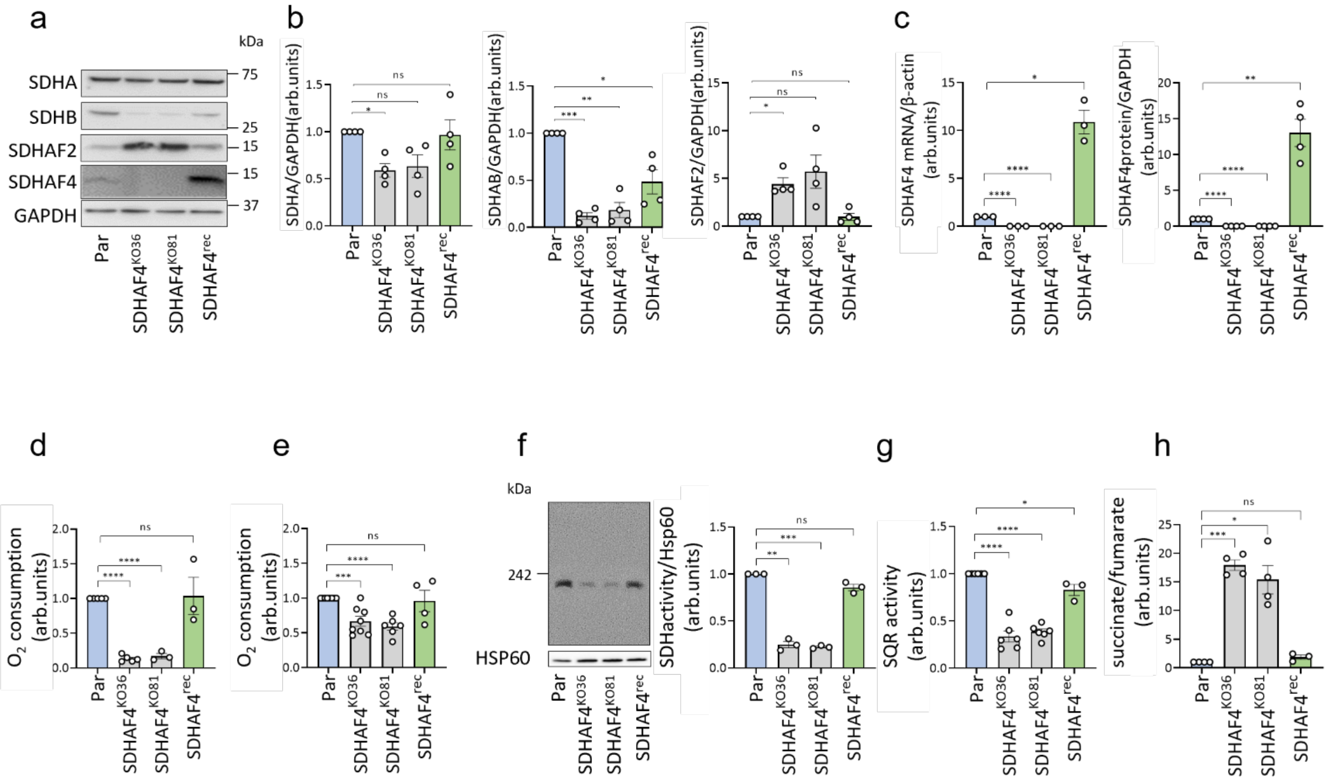

Supplementary Figure 2. Validation of cell lines with perturbed SDHAF4. (a) Representative gel (n=4, independent experiments) showing levels of relevant CII subunits and assembly factors in duplicate SDHAF4^{KO} cell lines. Parental, SDHAF4^{KO36}, SDHAF4^{KO81}, and SDHAF4^{rec} cells were subjected to SDS-PAGE followed by western blotting using anti-SDHA, anti-SDHB, anti-SDHAF2, and anti-SDHAF4 IgG. As a loading control, GAPDH levels were measured using anti-GAPDH IgG. (b) Quantitation of individual proteins assessed by western blotting, normalized to the GAPDH loading control. Both SDHA and GAPDH levels are quantified in ImageJ as arbitrary units (arb. units) and indicated as a ratio. (c) Parental, SDHAF4^{KO36}, SDHAF4^{KO81}, and SDHAF4^{rec} cells were evaluated by (left) qRT-PCR for the level of SDHAF4 mRNA, normalized to }-actin mRNA and expressed as a ratio of arb. units for each (n = 3 biologically independent samples), or (right) by densitometry of the westerns in panel (a), with SDHAF4 normalized to the GAPDH loading control, both of which were quantified in arb. units (n = 4 biologically independent samples). d) – f) Parental, SDHAF4^{KO36}, SDHAF4^{KO81}, and SDHAF4^{rec} cells were assessed for (d), routine respiration (n = 5 for parental and SDHAF4^{KO36} cells and n =3 for SDHAF4^{KO81}, and SDHAF4^{rec} cells, with individual data points shown), (e) CII-dependent respiration (n = 8 for parental and SDHAF4^{KO36} cells, n = 6 for SDHAF4^{KO81}, and n = 4 for SDHAF4^{rec} cells, from biologically independent replicates with individual data points shown), (f) SDH activity shown by a representative Native-PAGE gel (n = 3 biologically independent replicates). The SDH activity was evaluated using high-resolution clear chromatography followed by in-gel activity. Densitometric evaluation indicates normalized SDH to HSP60 on the y-axis, each measured as arb. units, and is shown on the right. (g) SQR activity (n = 6 for parental, SDHAF4^{KO36}, SDHAF4^{KO81} and n = 3 for SDHAF4^{rec} cells, from biologically independent samples with individual data points shown). The y-axis shows activity relative to theparental strain and quantified as arb. units. (h) succinate/fumarate ratio (n = 4 for parental,

SDHAF4^{KO36}, SDHAF4^{KO81} and n = 3 for SDHAF4^{rec} cells from biologically independent samples with individual data points shown). Bar graphs show mean values ± SEM; *, p<0.05; **, p<0.01; ***, p<001; and ****, p<0.0001; n.s., non-significant. Source data are provided as a Source Data file.

The exact p-values are: (b) SDHA/GAPDH, par. vs. SDHAF4^{KO36} p=0.0106, par vs. SDHAF4^{KO81} p=0.0560, par. vs. SDHAF4^{rec} p=0.8496.; SDHB/GAPDH, par. vs. SDHAF4^{KO36} p<0.0001, par vs. SDHAF4^{KO81} p=0.0021, par. vs. SDHAF4^{rec} p=0.0276; SDHAF2/GAPDH, par. vs. SDHAF4^{KO36} p=0.0122, par vs. SDHAF4^{KO81} p=0.0726, par. vs. SDHAF4^{rec} p=0.9697 (c) SDHAF4 mRNA/μ-actin, par. vs. SDHAF4^{KO36} p<0.0001, par vs. SDHAF4^{KO81} p.0001, par. vs. SDHAF4^{rec} p=0.0155; SDHAF4 protein/GAPDH, par. vs. SDHAF4^{KO36} p<0.0001, par vs. SDHAF4^{KO81} p=<0.0001, par. vs. SDHAF4^{rec} p=0.008 (d) parental vs. SDHAF4^{KO36} p<0.0001, parental vs. SDHAF4^{KO81} p<0.0001 , parental vs. SDHAF4^{rec} n.s. (e) parental vs. SDHAF4^{KO36} p<0.0001, parental vs. SDHAF4^{KO81} p=0.0003, parental vs. SDHAF4^{rec} p=0.7915; (f) parental vs. SDHAF4^{KO36} p=0.0022, parental vs. SDHAF4^{KO81} p=0.0002, parental vs. SDHAF4^{rec} p=0.0504; (g) parental vs. SDHAF4^{KO36} p<0.0001, parental vs. SDHAF4^{KO81} p<0.0001, parental vs. SDHAF4^{rec} p=0.0504; (g) parental vs. SDHAF4^{KO36} p=0.0003, parental vs. SDHAF4^{KO81} p=0.01, parental vs. SDHAF4^{rec} p=0.1523

Supplementary Figure 3

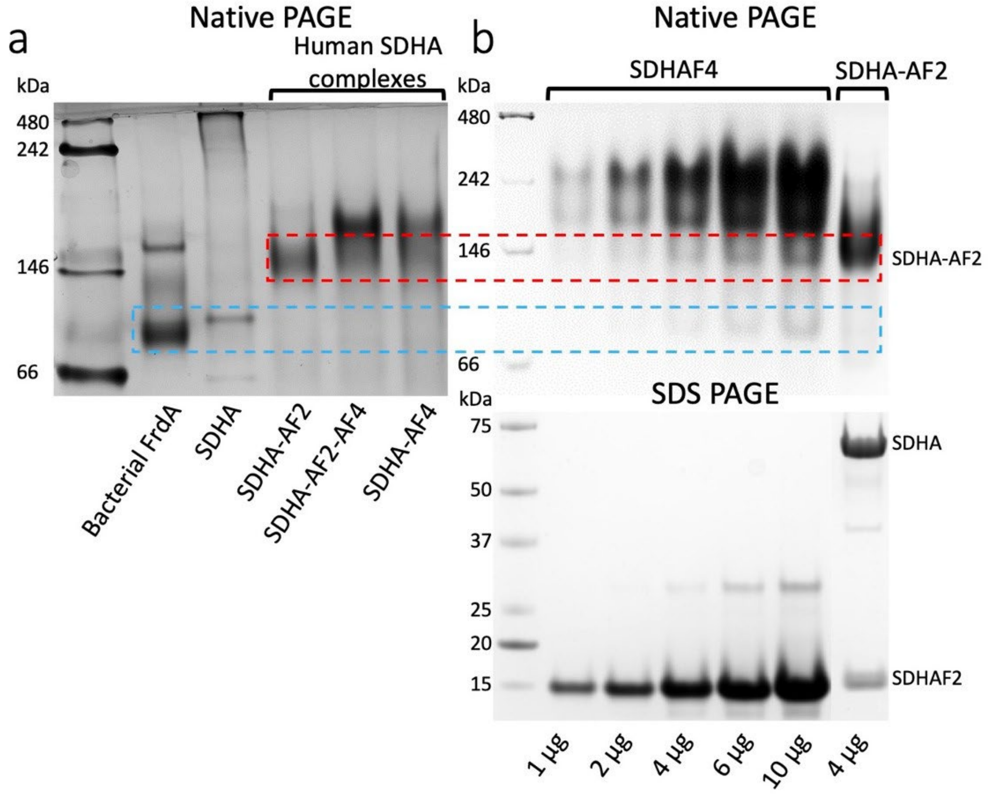

Supplementary Figure 3. Identification of molecular species consistent with the ~100 kDa and ~150 kDa bands that accumulate in knock-out strains. (a) Migration of purified SDHA, SDHA-AF2, SDHA-AF2-AF4, and SDHA-AF4 on a 10% native PAGE. SDHAF4-containing complexes migrate slower than the SDHA-AF2 complex. Note, gels used here to separate purified proteins were 10% native PAGE while those used to separate cell lysates were gradient. Because of this, the relative positions of the bands can be inferred, but the migration will differ. (b) SDHAF4 oligomerization on both native and SDS gels at concentrations between 0.5 – 5 mg/ml. The migration of SDHA-AF2 (2mg/ml) is shown for comparison. Comparison of the native gels in (a) and (b) identifies that purified SDHAF4 forms oligomers that can migrate at a molecular weight similar to SDHA-AF2 complex (red box) and isolated SDHA (blue box). The SDS-PAGE gel shows that SDHAF4 forms dimers at higher protein concentrations even in denaturing conditions. Both (a) and (b) are representative of n = 3 independent protein preparations. Source data are provided as a Source Data file.

Supplementary Figure 4

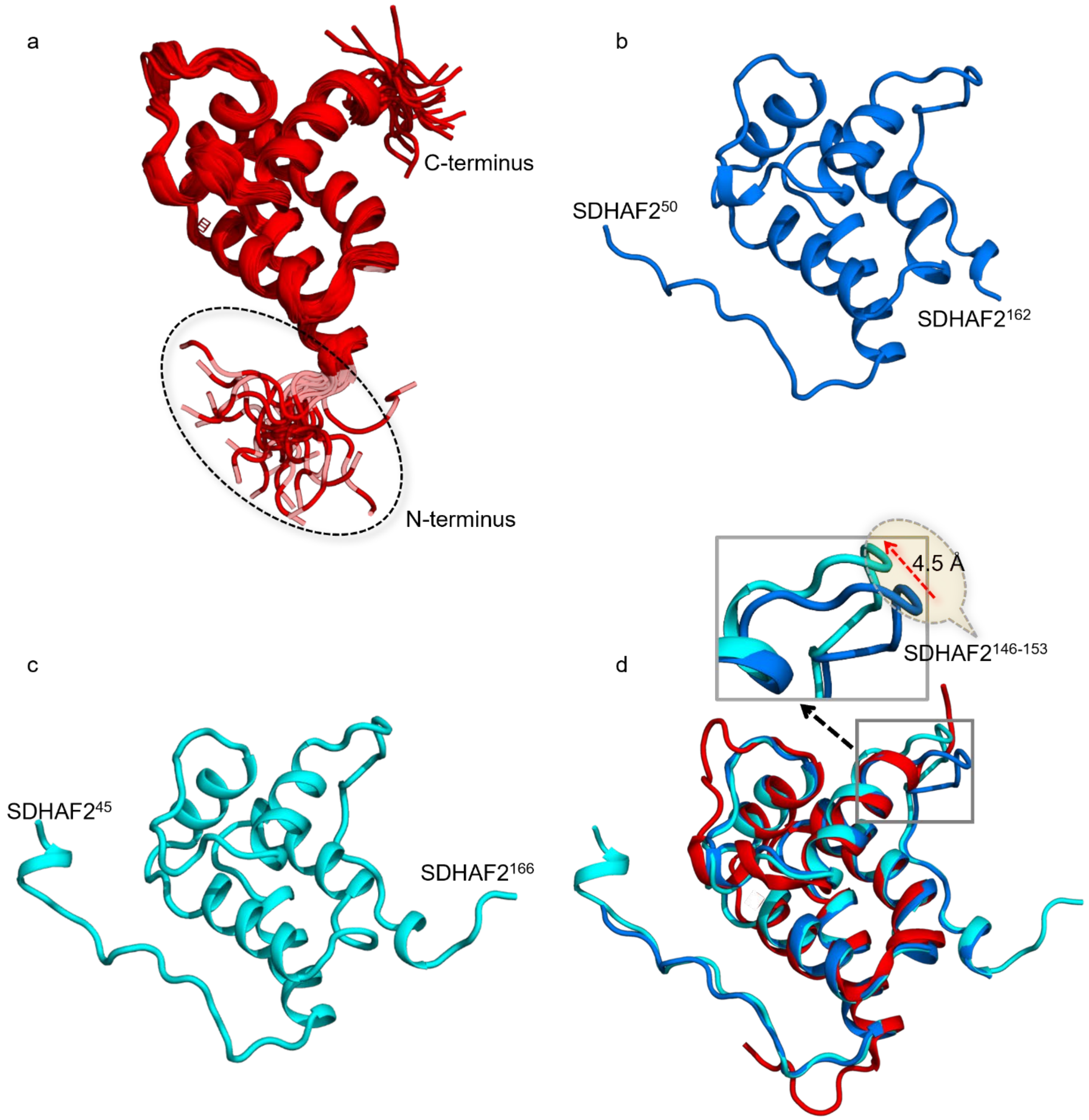

Supplementary Figure 4. Changes in disorder in SDHAF2 upon binding to SDHA. (a) NMR structure of the yeast SDHAF2 homolog (PDBID 2LM4)[1]; all 20 deposited structures are shown. Both the N- and C-termini exhibit intrinsic disorder. (b) Human SDHAF2 resected from the SDHA-AF2 structure (PDB ID 6VAX)[2]. The termini of SDHAF2 when bound to SDHA increase in ordering but lack secondary structure. Full-length SDHAF2 contains residues 1 – 166, and residues are still disordered at both termini. (c) SDHAF2 resected from the SDHA-AF2-AF4 structure, reported here. Additional residues at each of the termini gain order compared to SDHA-AF2 structure. The entire C-terminus is now ordered. (d) Comparison of the structures of the isolated yeast SDHAF2 homolog (*red*, single state from 20 deposited structures), human SDHAF2 resected from the SDHA-AF2 structure (*blue*), and human SDHAF2 resected from the SDHA-AF2-AF4 structure (*cyan*). Differences are observed in the changes in order at the termini and in residues SDHAF2$^{146\text{-}153}$ (*inset*). Note that the termini of the isolated yeast SDHAF2 homolog are disordered, while those of the bound SDHAF2 are ordered, but lack secondary structure.

Supplementary Figure 5

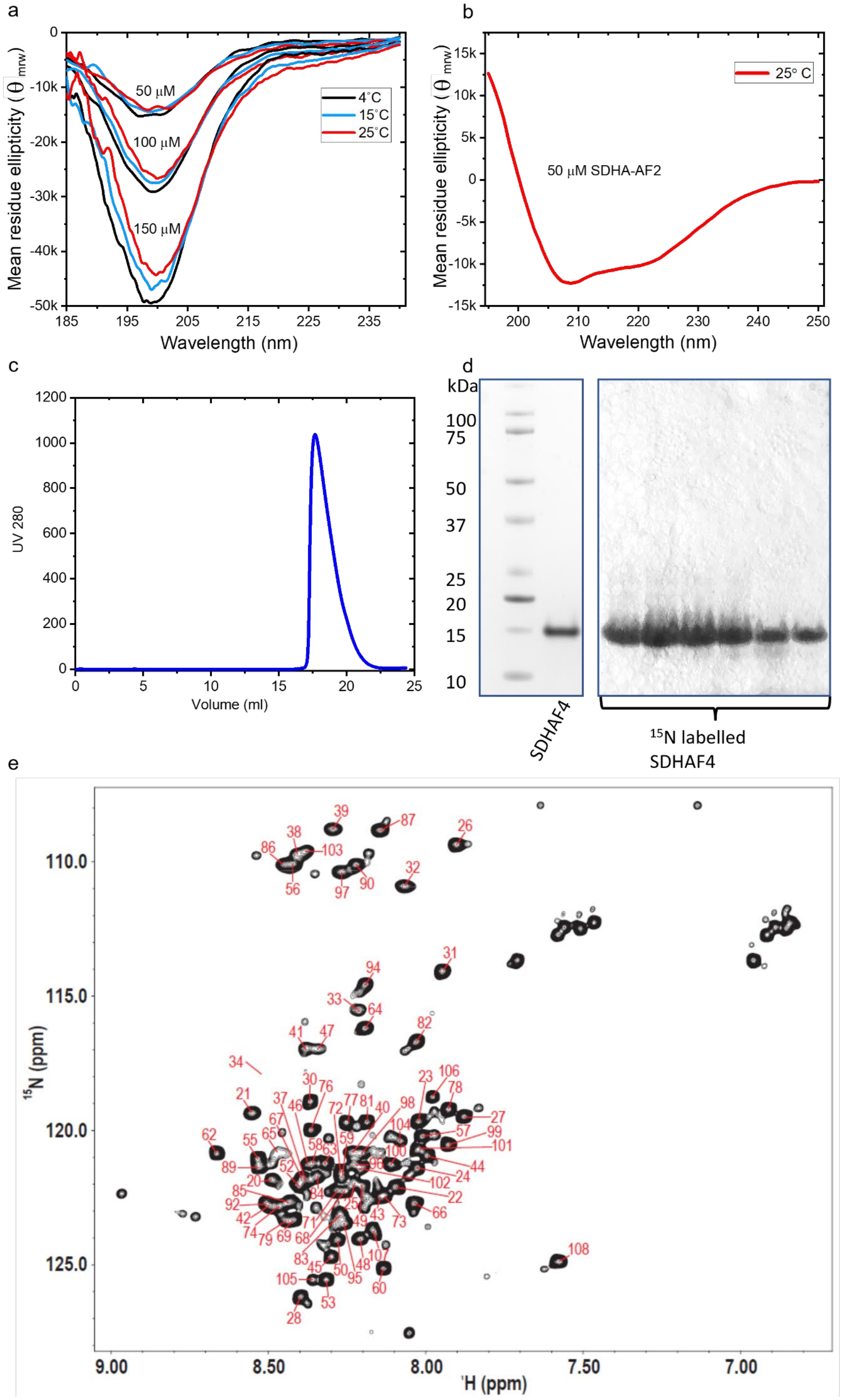

f

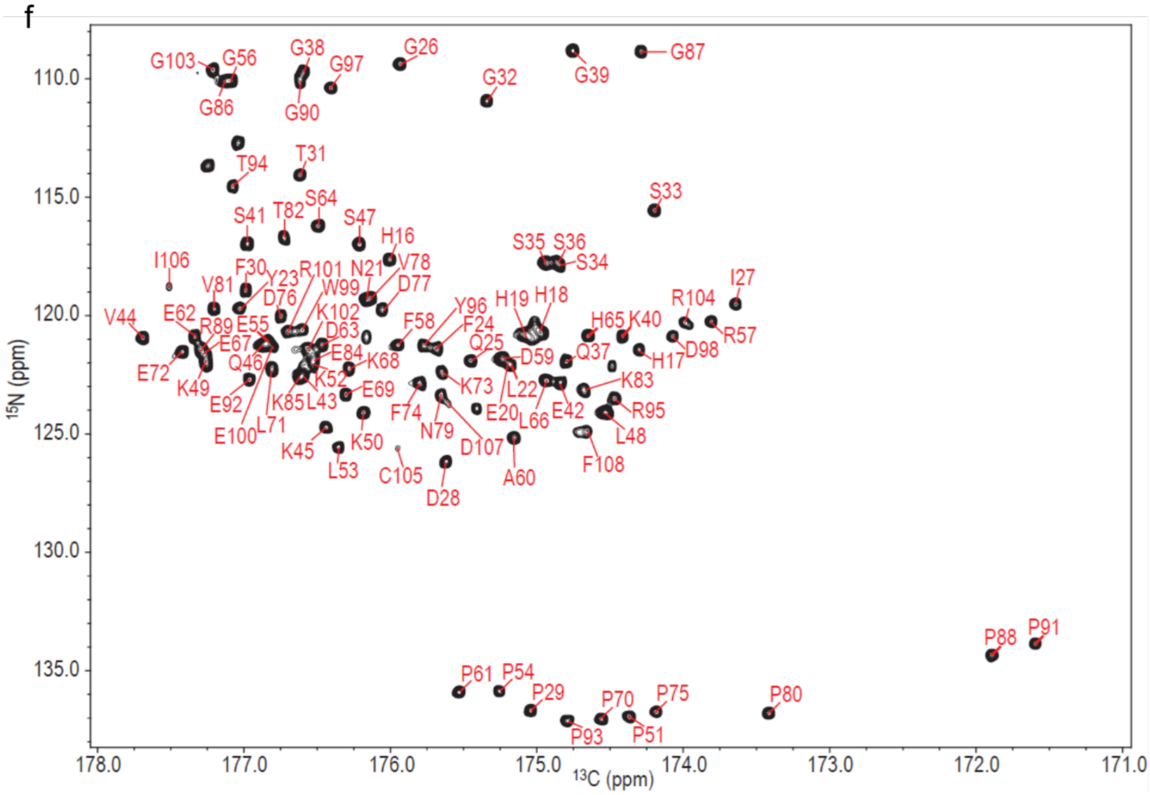

15N (ppm)
13C (ppm)
110.0
115.0
120.0
125.0
130.0
135.0
178.0
177.0
176.0
175.0
174.0
173.0
172.0
171.0

g

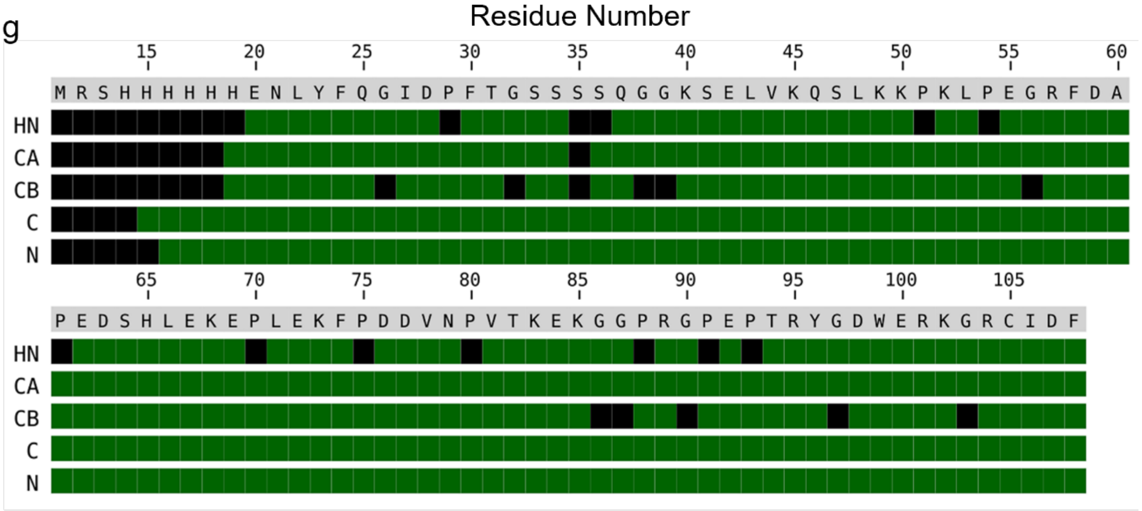

Residue Number
M R S H H H H H H E N L Y F Q G I D P F T G S S S S Q G G K S E L V K Q S L K K P K L P E G R F D A
P E D S H L E K E P L E K F P D D V N P V T K E K G G P R G P E P T R Y G D W E R K G R C I D F
HN
CA
CB
C
N

h

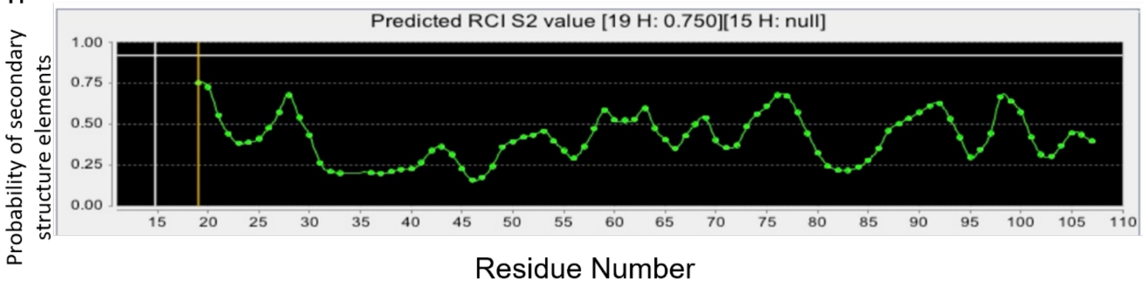

Predicted RCI S2 value [19 H: 0.750][15 H: null]
Probability of secondary structure elements
Residue Number

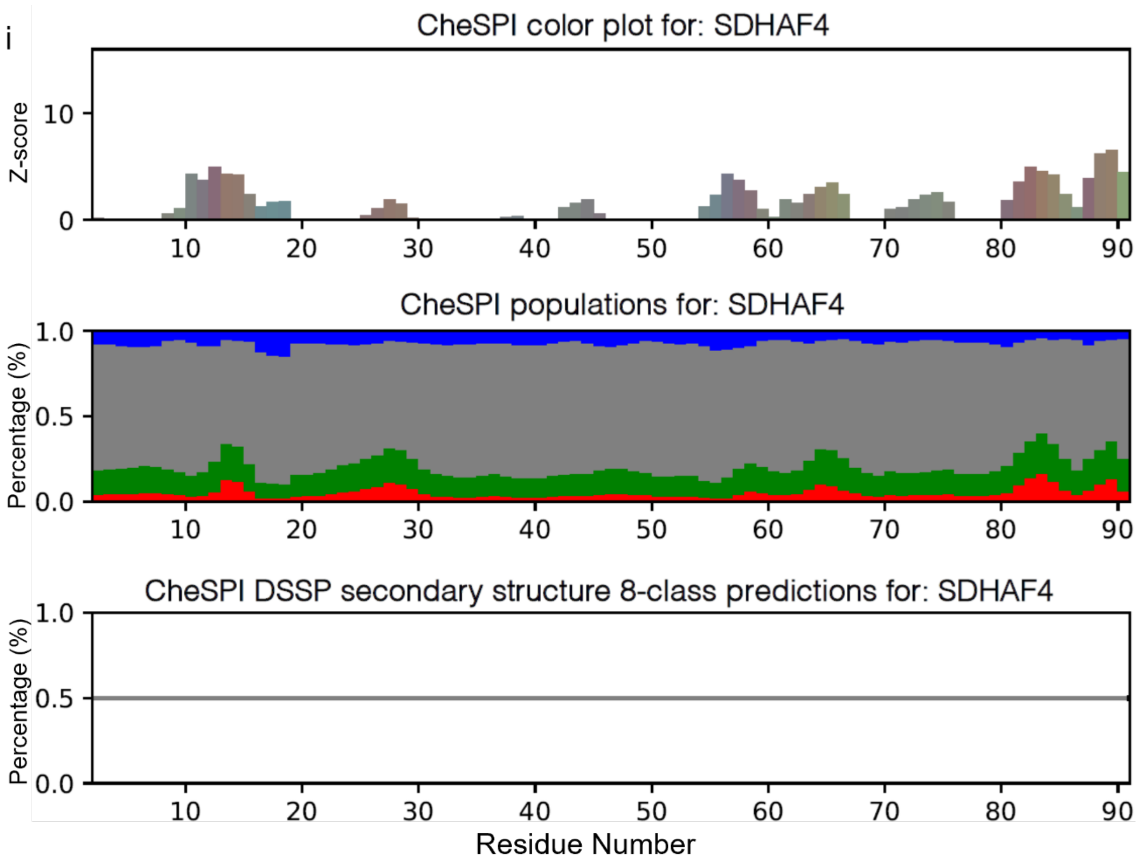

Supplementary Figure 5. Isolated SDHAF4 is an intrinsically disordered protein. (a) Circular dichroism spectra of SDHAF4 collected at three temperatures (4 °C, 15 °C and 25 °C) and three protein concentrations (50 µM, 100 µM, and 150 µM) all show a peak minimum at 200 nm, suggesting that SHDAF4 is an intrinsically disordered protein. (b) To demonstrate how a typical CD spectrum of a folded protein differs from the spectrum of SDHAF4, we used the SDHA-AF2 complex (50 µM) as a comparator and collected the CD spectrum at 25 °C. The folded SDHA-AF2 protein exhibits minima at 208 and 222 nm and at 218 nm that reflect the presence of α-helices and β-strands, respectively. These features are absent in the spectrum of SDHFA4. (c) Size exclusion chromatography profile of ^{15}N labeled His-SDHAF4 used for NMR experiments. The y-axis shows absorbance at 280 nm (A_{280}) expressed as milli Absorbance Units (mAU). (d) Following size exclusion chromatography, the purified ^{15}N labeled protein was separated by SDS-PAGE gel (right) and compared to unlabeled SDHAF4 (left) to confirm the purity. (e) Full ^{15}N-^{1}H HSQC spectrum with assigned peaks transferred from the assignments of the carbon direct detect spectra via the HNCO. The N-terminal His- tag, SDHAF4$^{35-36}$, and sidechain as well as peaks from a putative minor conformation were not assigned. SDHAF4^{34} is a very weak peak and only shows at very low contour levels. The assignment for SDHAF4$^{20-21}$, SDHAF4^{34,35} and SDHAF4$^{104-107}$ suffered from heavy overlap or low peak intensity. (f) Assigned carbon direct detect CON spectrum serving as the bases for the amide-based assignment. (g) Assignment Panel (NMRViewJ). Green squares indicate assigned, black un-assigned atoms. Proline and glycine residues serve as a checkpoint in the carbon direct detect methods. They have black squares as they either do not contain Cb (Gly) or NH (Pro). SDHAF4-S35, S36 could not be fully assigned as these residues are expected to have little to no chemical shift dispersion and therefore overlap, all other residues have been fully assigned. (h) Secondary structure assignment panel based on the Talos+ calculation[3]. The y- axis shows the fractional probability of the

existence of secondary structure using the order parameter, S^2. This indicates high flexibility and therefore a low probability of secondary structure elements. These S^2 order parameters are derived from the chemical shifts as described by Berjanskii and Wishart[4]. Talos+ calculations for secondary structure predictions are based on differences between the actual chemical shifts and tri-peptide reference shifts using an artificial neural network for best prediction[3]. (i) Secondary structure assignment using chemical shift secondary structure population inference (CheSPI)[5] based analysis to measure local structure and disorder. (Top) CheSPI Z-score representation uses RGB color to define the structural components in the proteins where blue indicates sheets, red indicates helices, green indicates turns, and grey with principal components close to zero indicates disorder. (Middle) stacked bar plot of CheSPI populations in percentage (%) of extended (blue), helical (red), turn (green), and non-folded (grey). (Bottom) Cartoon of the most confident CheSPI prediction of eight class DSSP secondary structure in percentage (%), using red lines for α- helices (H), magenta for 3_{10}-helices (G), blue arrows for sheets (E), and bridges (B), green arcs for turns (T), and grey and black lines for coils (C) and bends (S). This orthogonal analysis is consistent with the assignment of SDHAF4 as an intrinsically disordered protein. Source data are provided as a Source Data file.

Supplementary Figure 6

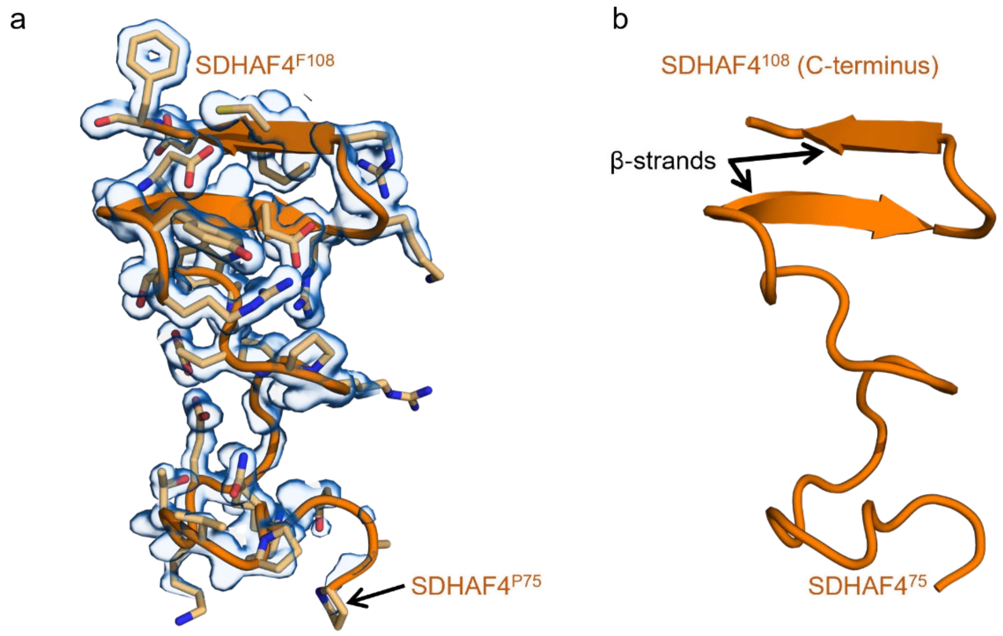

c

```
H. Sapiens          ------MTPSRLPWL----------LSWVSATAWRAARS--PL-LCHSLRKTS---S-SQ  37
R. sphaeroides      ------------------------------------------------------------  0
S. cerevisiae       MLCAIKSTGYRYPRTGALNLLRGRPFNMATRKITTERIPGPPKLPREEQEEFERLQRIAT  60
D. melanogaster     MQSVTRQTARVLPQVGK------QVSYLSTSGAWRASAGGSDM-VVEIKEPK------T-  46
A. thaliana         --------------------------MATNNIVRLIAPSRSV-SSRFLEPVS---RFLS  29
D. rerio            ------------------------------------------M-NTAGLRPAG---Y-A-  12
G. gallus           --------------------------MALRLLRAAARS--SL-PCSSVRSY------TS  24

H. Sapiens          GGKSE-----L-----VKQSLKKPKLPEGRFDAP-ED--SHLEKEPLEKFPDDVNPVTKE  84
R. Sphaeroides      ----------M-----TE--ETRKDLPPEALRALAEA--E------ERRRQAKALDLPKE  35
S. cerevisiae       SQEAIDQYNAQATADRTKESLNSPLLTKNDIGSF-----SPEFSKTIPEFEGDVNPKTGE  115
D. melanogaster     RTEKL-----M-----AFQKKLRAKTPLGKLDEFSRH--PYQEKEPLKPWPNQTNPYTGE  94
A. Thaliana         SGTPP-----PPQAPSPNQDLNRDVKPDQNLQQNLQMQKEEEEEGEGGGGGEFVNEDTGE  84
D. rerio            SGGVT-----K-----DKEPLKKAKTPQGRFDME-ET--ESKSKDVLERFPDDVNPETKE  59
G. gallus           EGRPE-----P-----AKQTLKKPKLPVGRFDEP-KE--SNIEREPLEKFPDGINPATKE  71
                                                  :                            *

H. Sapiens          KGGPRGPEPTRYGDWERKGRCIDF      108
R. Sphaeroides      IGGRNGPEPVRFGDWEKKGIAIDF      59
S. cerevisiae       VGGPK-QDPLRHGDYSFNGRVTDF      138
D. melanogaster     IGGPAGPEPTRYGDWERKGRVSDF      118
A. Thaliana         IGGPRGPEPTRYGDWEQRGRCSDF      108
D. rerio            KGGPRGPEPTRYGDWERKGRCIDF      83
G. gallus           RGGPKGPEPTRFGDWERKGRCIDF      95
                     **    :* *.**:. .*   **
```

Supplementary Figure 6. Fold and sequence conservation of SDHAF4. (a) Structure of SDHAF4 resected from the SDHA-AF2-AF4 complex shown with side chains and superimposed onto a 2|F_o|-|F_c| electron density map (blue, at 1σ) calculated following molecular replacement but before building the SDHAF4 polypeptide. Only residues SDHAF4$^{75\text{-}108}$ were observed in the context of the SDHA-AF2-AF4 complex. (b) Ribbon representation of the SDHAF4 structure (residues 75-108) resected from the SDHA-AF2-AF4 structure. (c) Sequence alignment of SDHAF4 homologs. Comparators are homologs from human, *Rhodobacter sphaeroides, Saccharomyces cerevisiae* (yeast)*, Drosophila melanogaster* (fruit fly)*, Arabidopsis thaliana* (plant)*, Danio rerio* (zebrafish)*, Gallus gallus* (chicken). Red boxes highlight conserved residues that were selected for mutation.

Supplementary Figure 7

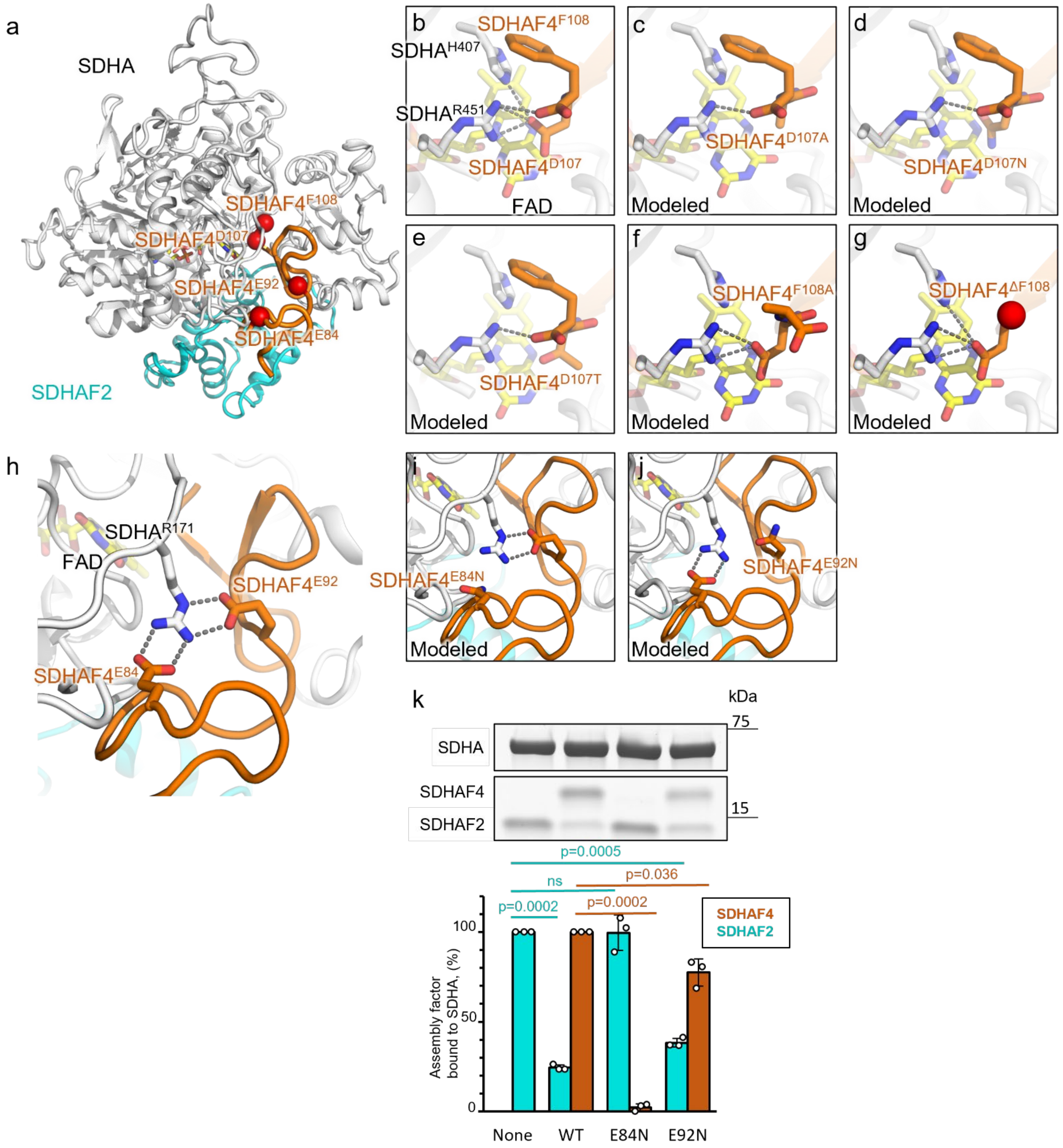

Supplementary Figure 7. Mutational analysis of SDHAF4. (a) Locations of substitution and truncation mutations of SDHAF4 in the context of the SDHA-AF2-AF4 crystal structure. The affected residues included SDHAF4^{E84}, SDHAF4^{E92}, SDHAF4^{D107}, and SDHAF4^{F108}. (b) Interactions between the SDHAF4 C-terminus and the SDHA active site. SDHAF4^{D107} and SDHAF4^{F108} interact with the active site of SDHA including the substrate-interacting residues SDHA^{H407} and SDHA^{R451}. (c-g), Models of the SDHAF4 C-terminal residue mutations. Coot [6] was used to substitute residues. In each case, the most favored rotamer that made reasonable interactions was hand selected. (c) SDHAF4^{D107A}, (d) SDHAF4^{D107N}, (e) SDHAF4^{D107T}, (f) SDHAF4^{F108A} and (g) SDHAF4$^{\}F108}$. (h) detailed interactions between SDHAF4^{E84} and SDHAF4^{E92} with the conserved

SDHA^{R171}. (i-j). Modeling of (i), SDHAF4^{E84N} and (j) SDHAF4^{E92N} predicts the disruption of stabilizing interactions between SDHA and SDHAF4. (k) Displacement of SDHAF2 from the SDHA-AF2 complex by SDHAF4^{E84N} and SDHAF4^{E92N} showed that SDHAF4^{E84N} did not bind, while SHDHAF4^{E92N} bound but was ~40-50% less efficient. The SDS-PAGE gel is representative of n=3 independent experiments. We quantified the amount of either SDHAF2 (brown) or SDHAF4 (teal) as compared to SDHA by using ImageJ quantitation in arbitrary units and expressed these as a percentage. The y-axis shows what percentage of SDHA has each assembly factor bound when wild-type or mutant SDHAF4 is used. The bars show mean values ± SD, statistics were calculated by paired two-tailed Student's t-test. Source data are provided as a Source Data file.

Supplementary Figure 8

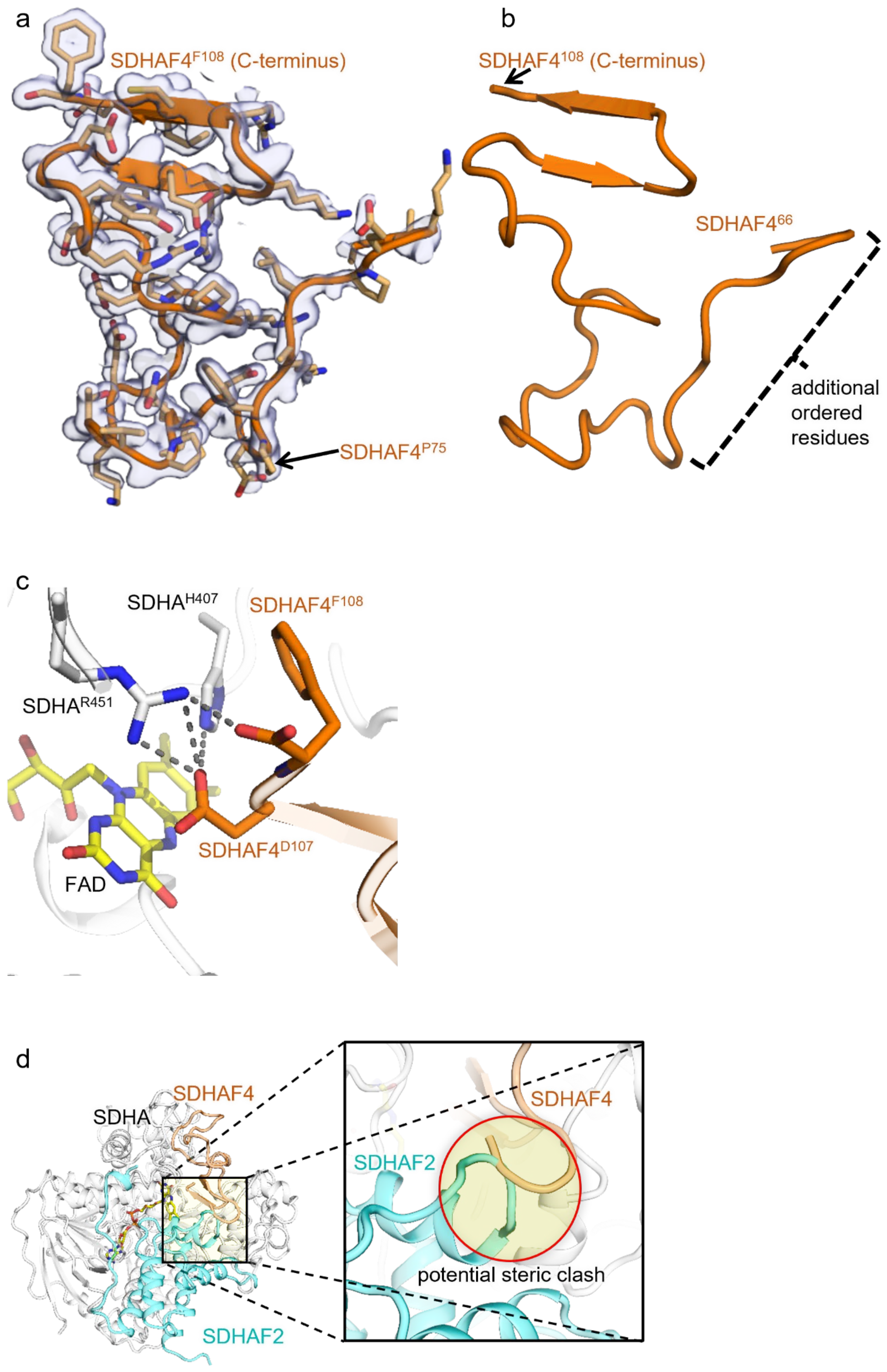

Supplementary Figure 8. SDHAF4 in the context of the SDHA-AF4 complex. (a) SDHAF4 (*orange*) resected from the SDHA-AF4 complex and superimposed onto a $2|F_o|-|F_c|$ electron density map contoured at 1σ level (*blue*). An additional nine residues of SDHAF4 are ordered in SDHA-AF4 compared to SDHA-AF2-

AF4 complex. In the newly ordered region, a tight turn is facilitated by SDHAF4^{P75}; mutation of SDHAF4^{P75} is associated with vagal paraganglioma [7]. (b) Ribbon representation of the SDHAF4 structure (residues 66-108) resected from the SDHA-AF4 structure. (c) Interactions between the active site of SDHA and the C-terminus of SDHAF4 appear to be unchanged when compared to the SDHA-AF2-AF4 structure. (d) Superposition of SDHA-AF2-AF4 and SDHA-AF4 highlight region that the additionally ordered regions of SDHAF4 in the SDHA-AF4 structure would be steric conflict with SDHAF2 if it remained bound (*inset*, red circle).

Supplementary Figure 9

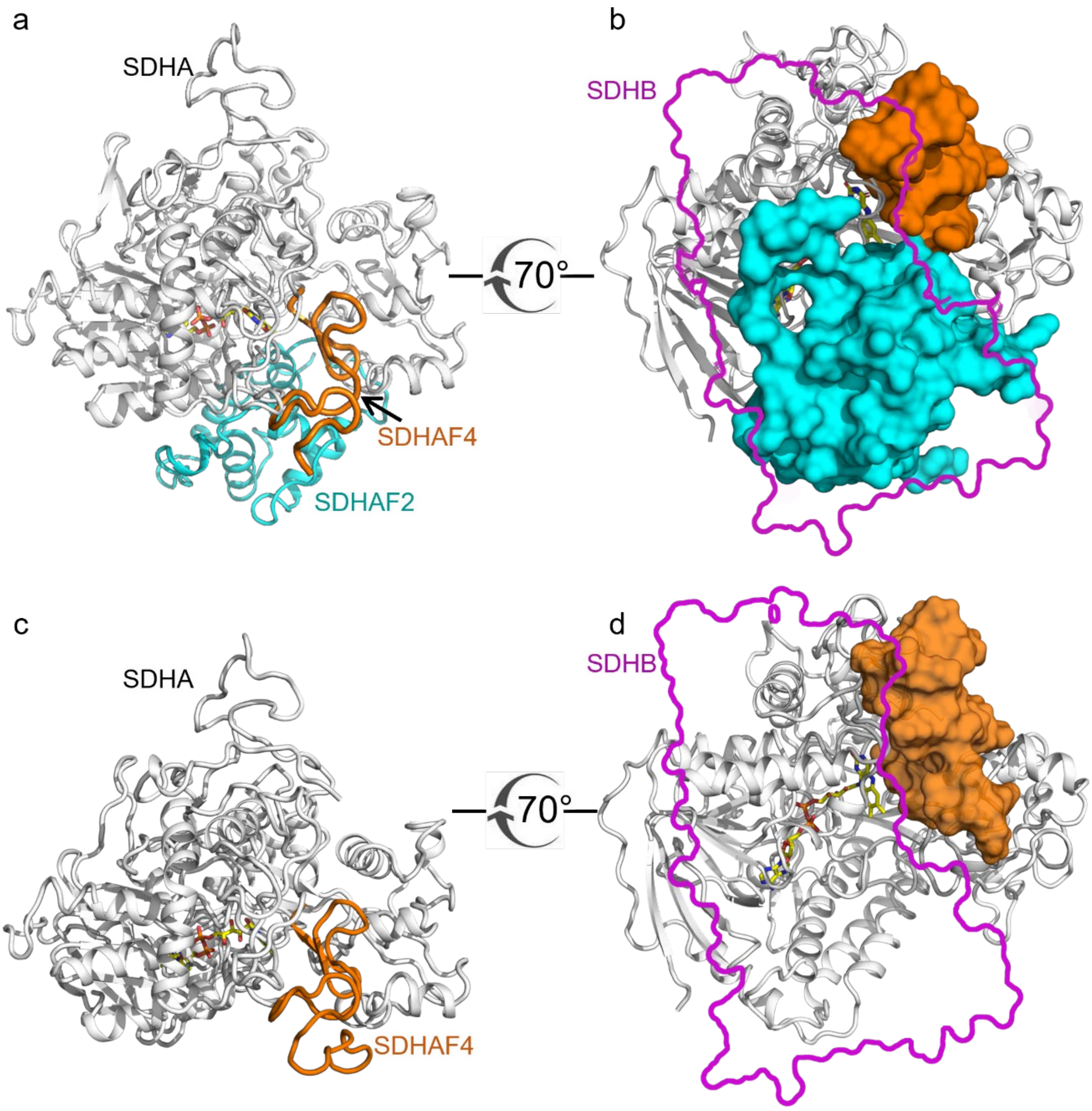

Supplementary Figure 9. The footprint for SDHB on SDHA overlaps with the binding sites for both the SDHAF2 and the SDHAF4 assembly factor. SDHA is shown in *white*, SDHAF2 is shown in *cyan*, and SDHAF4 is shown in *orange*. The footprint where SDHB binds is shown as a pink outline. (a) The structure of the SDHA-AF2-AF4 complex is shown as ribbons. (b) A rotated view shows SDHAF2 and SDHAF4 as surfaces with the binding location for SDHB shown as an outline. The location of SDHB was identified by the superposition of the porcine CII structure PDB ID 3SFD[8] onto the structure of SDHA-AF2-AF4. (c) The structure of the SDHA-AF4 complex is shown as ribbons. (d) A rotated view of the SDHA-AF4 complex with SDHAF4 is shown as a surface and the binding location for SDHB is shown as an outline. The location of SDHB was identified by the superposition of the porcine CII structure PDB ID 3SFD[8] onto the structure of SDHA-AF4.

Supplementary Supplementary Figure10

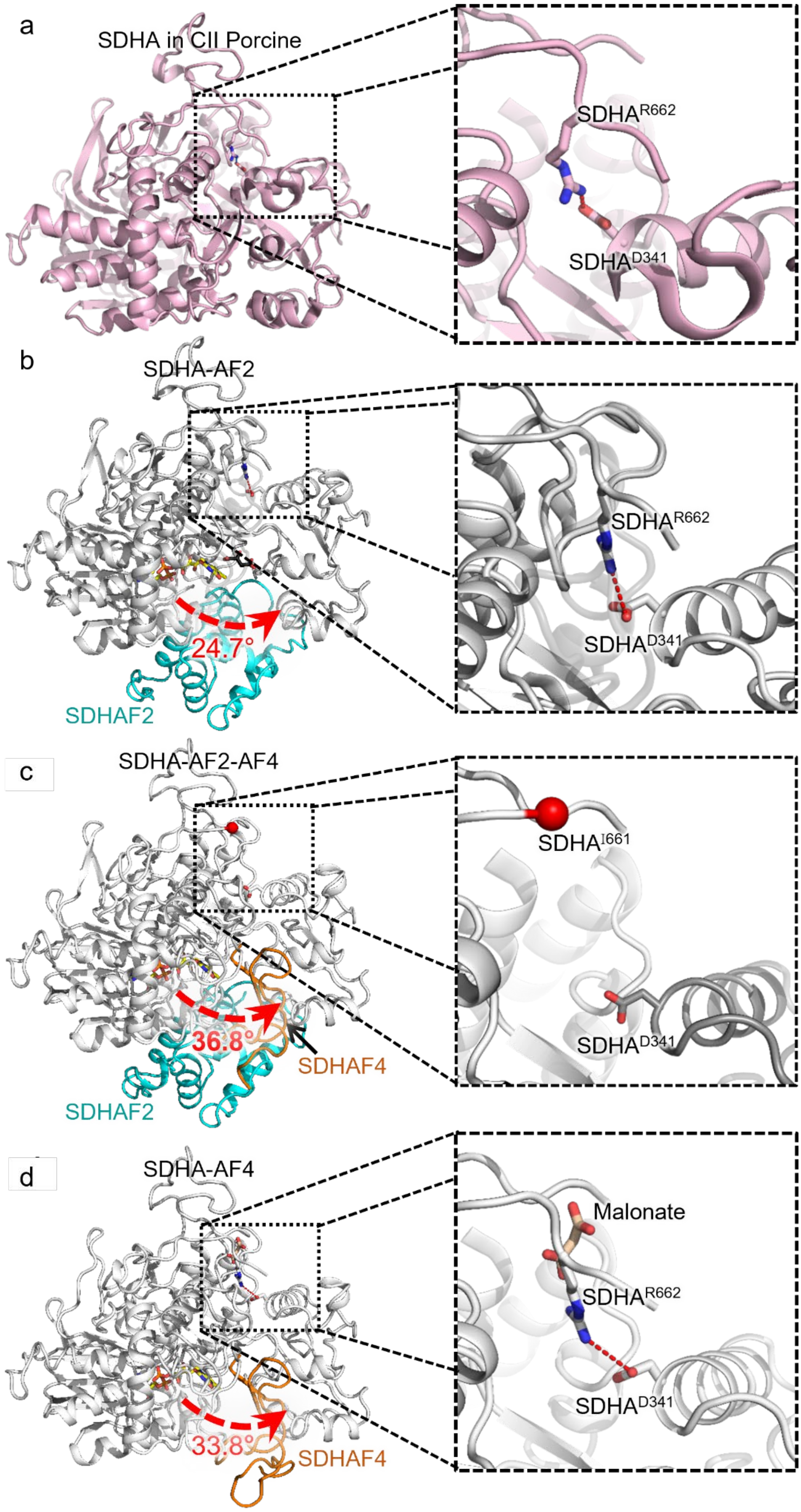

Supplementary Figure 10. Changes in disorder of SDHA during maturation. Porcine SDHA is shown in *pink*, human SDHA is shown in *white*, human SDHAF2 is shown in *cyan*, and human SDHAF4 is shown in *orange.* The differences in the interdomain angle between the SDHA flavin binding and capping domains

when bound to assembly factors versus SDHA in assembled complex II (PDB ID 3SFD[8]) are indicated in *red.* (a) The structure of SDHA in the context of mature CII (SDHABCD) has been determined from porcine CII (95% identical and 96% similar to human SDHA). A notable salt bridge between residues SDHA^{R662} and SDHA^{D341} stabilizes the positions of two domains that surround the active site (PDB ID 3SFD[8]). (b) The interaction between SDHA^{R662} and SDHA^{D341} is also present in SDHA-AF2 complex (PDB ID 6VAX)[2]. (c) However, this salt bridge is disrupted in SDHA-AF2-AF4 complex. As a result, the C-terminal residues of SDHA become disordered after SDHA^{I661} (*red* sphere) and lack associated electron density in the crystal structure. (d) This interaction is restored in the SDHA-AF4 complex.

Supplementary Figure 11

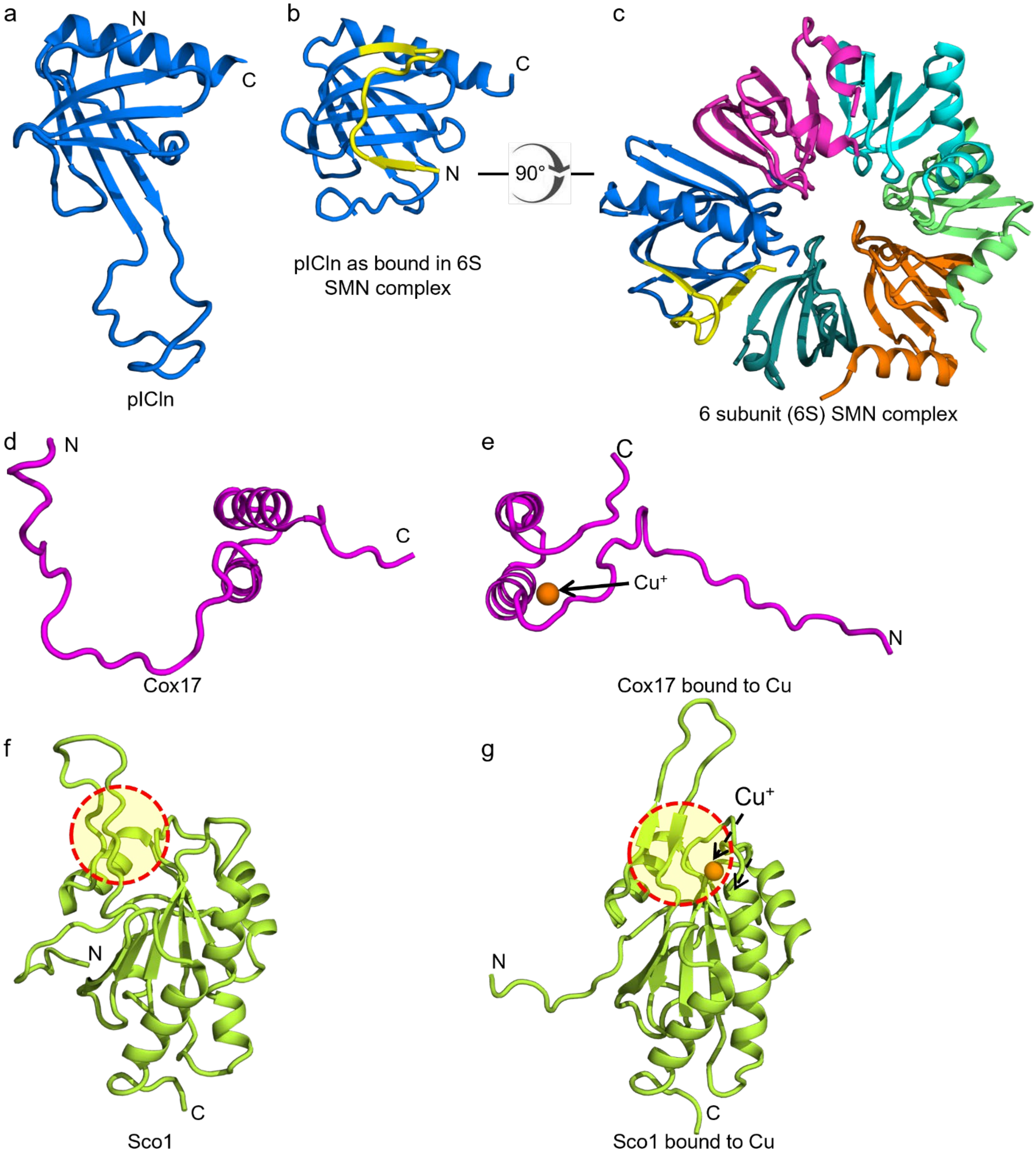

Supplementary Figure 11. Examples of changes in disorder of assembly factors during biogenesis. (a) pICln (blue ribbon) is an assembly chaperone for small nuclear ribonucleoproteins. pICln is disordered at its N-terminus as an isolated protein (PDB ID 1ZYI)[9] and contains a long, unstructured loop. **(b)** pICln upon binding to the SMN complex (PDB ID 4F7U)[10] the N-terminus of pICln organizes into a β-strand (yellow ribbon). **(c)** SMN complex with six subunits. The decrease in disorder of the N-terminus of pICln probably stabilizes the assembly intermediate[9,10]. **(d)** Cox17 is an assembly factor for respiratory complex IV and changes from open conformation (violet ribbon, PDB ID 1U97)[11] to a **(e)** closed conformation (PDB ID

1U96)[11] upon binding to copper[11] (orange sphere). **(f)** Sco1 is involved in the delivery of copper to complex IV. A core loop (red dotted circle) of Sco1 becomes ordered when the isolated polypeptide (lemon ribbon, PDB ID 2GT5)[12] **(g)** binds to the metal ions (Cu as orange sphere, PDB ID 2GQM)[12].

Supplementary Figure 12

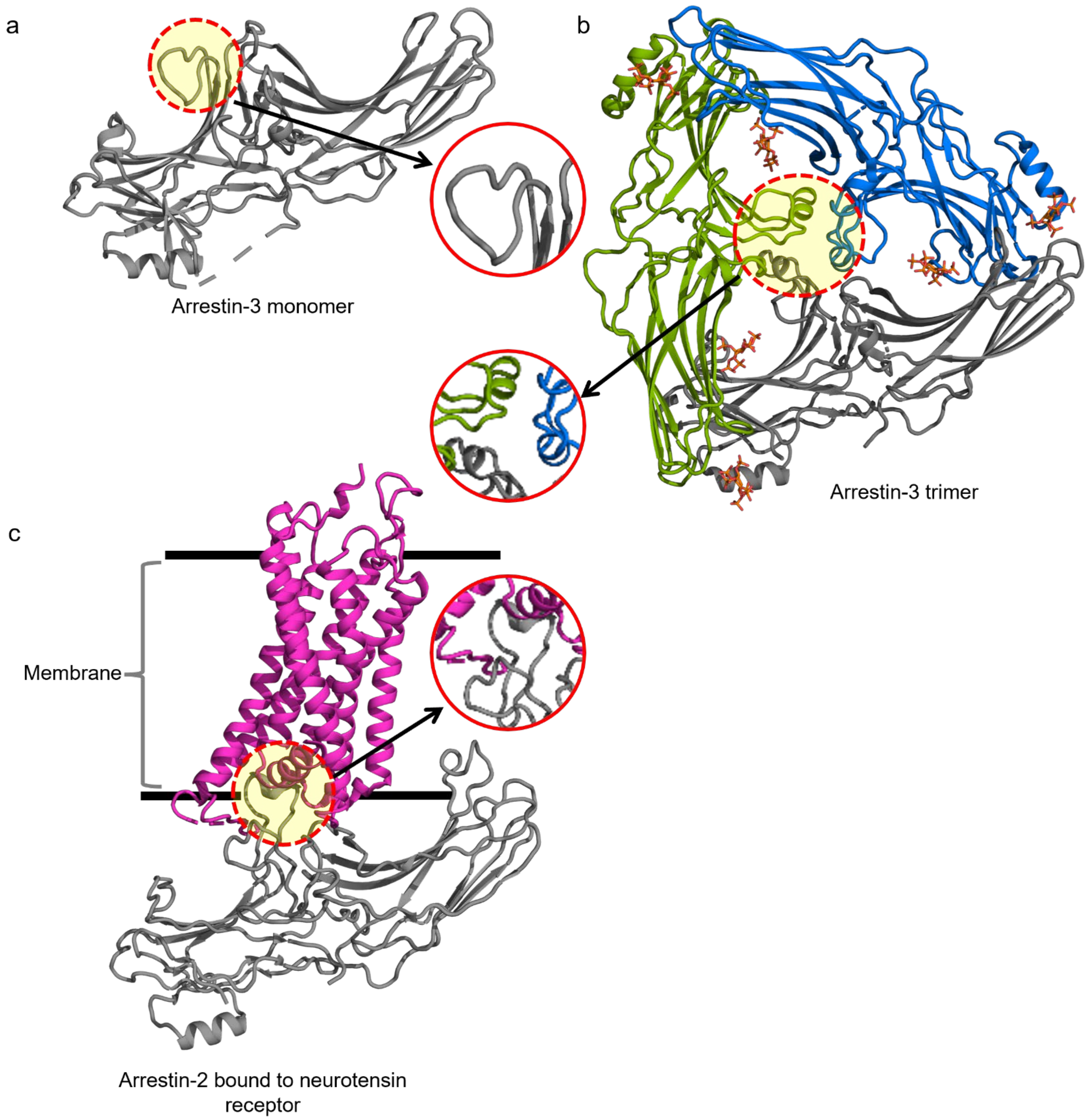

Supplementary Figure 12. Changes in disorder in the protomers of self-assembling arrestin complexes. (a) A mammalian signal-transducing protein called arrestin-3 is normally a monomer in solution (grey ribbon, left, PDB entry 3P2D)[13]. An unstructured loop (red dotted circle) is involved in direct interactions with >800 distinct binding partners. (b) Interaction with the small molecule inositol hexaphosphate can induce arrestin-3 trimerization (PDB ID 5TV1)[14]. In this trimer, the unstructured region of monomeric arrestin orders into an α-helix that mediates trimerization (red dotted circle). (c) Structures of the arrestin-2 homolog with the neurotensin receptor (PDB ID 6UP7)[15], which belongs to the family of G protein-coupled receptors. The same region of arrestin that forms an α-helix during self-trimerization now forms a dynamic extended conformation

with receptor[15,16]. The plasticity of this region may assist in self-assembly and may promote binding to a broad range of partners.

Supplementary Figure 13

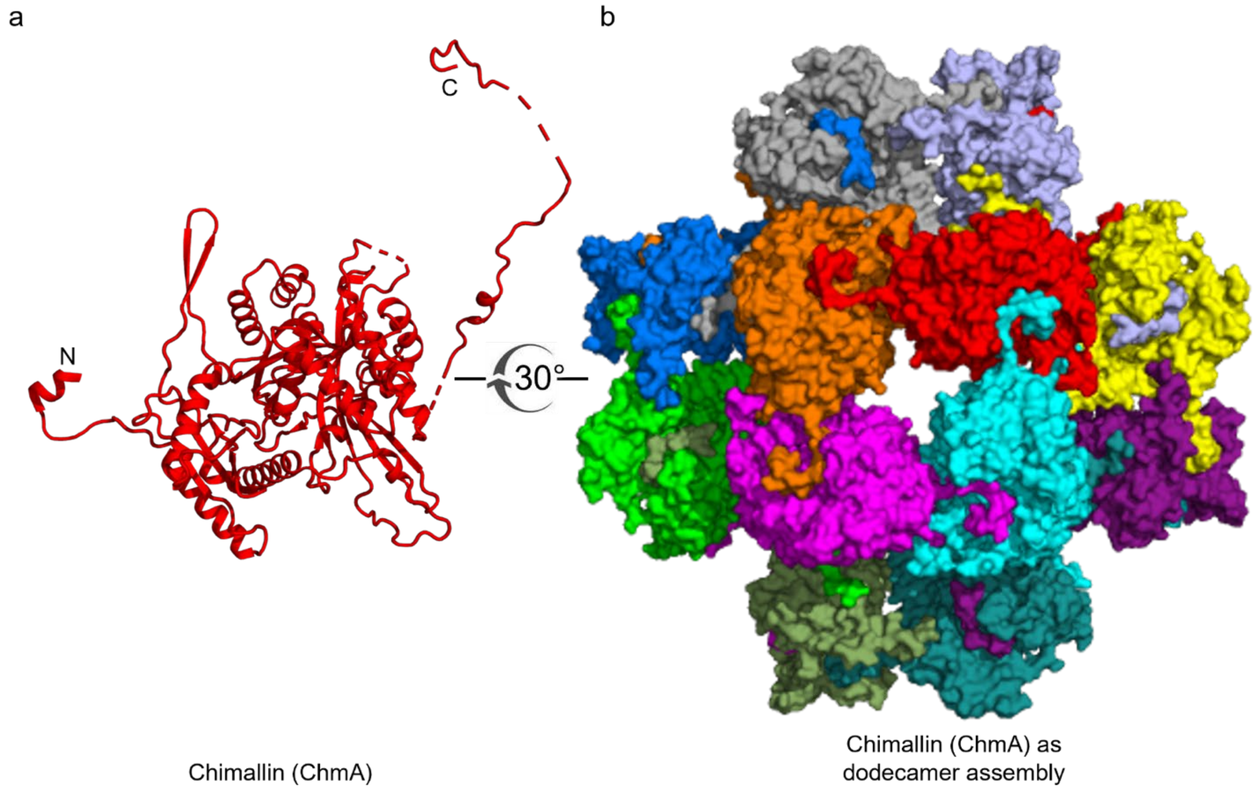

Supplementary Figure 13. Changes in disorder in the self-assembling chimallin complex. The chimallin protomer self-assembles into an extremely large structure that encapsulates the bacteriophage genome. The structure of chimallin has only been determined in the context of the nuclear shell. (a) A resected protomer of chimallin highlights the lack of structure at both the N- and C-termini (PDB ID 7SQS)[17]. (b) The termini of each chimallin protomer interact with adjacent molecules in the nuclear shell (PDB ID 7SQQ)[17]. The high level of disorder and lack of secondary structure may provide plasticity, which can allow the nuclear shell to assemble[17-19].

Supplementary Figure 14

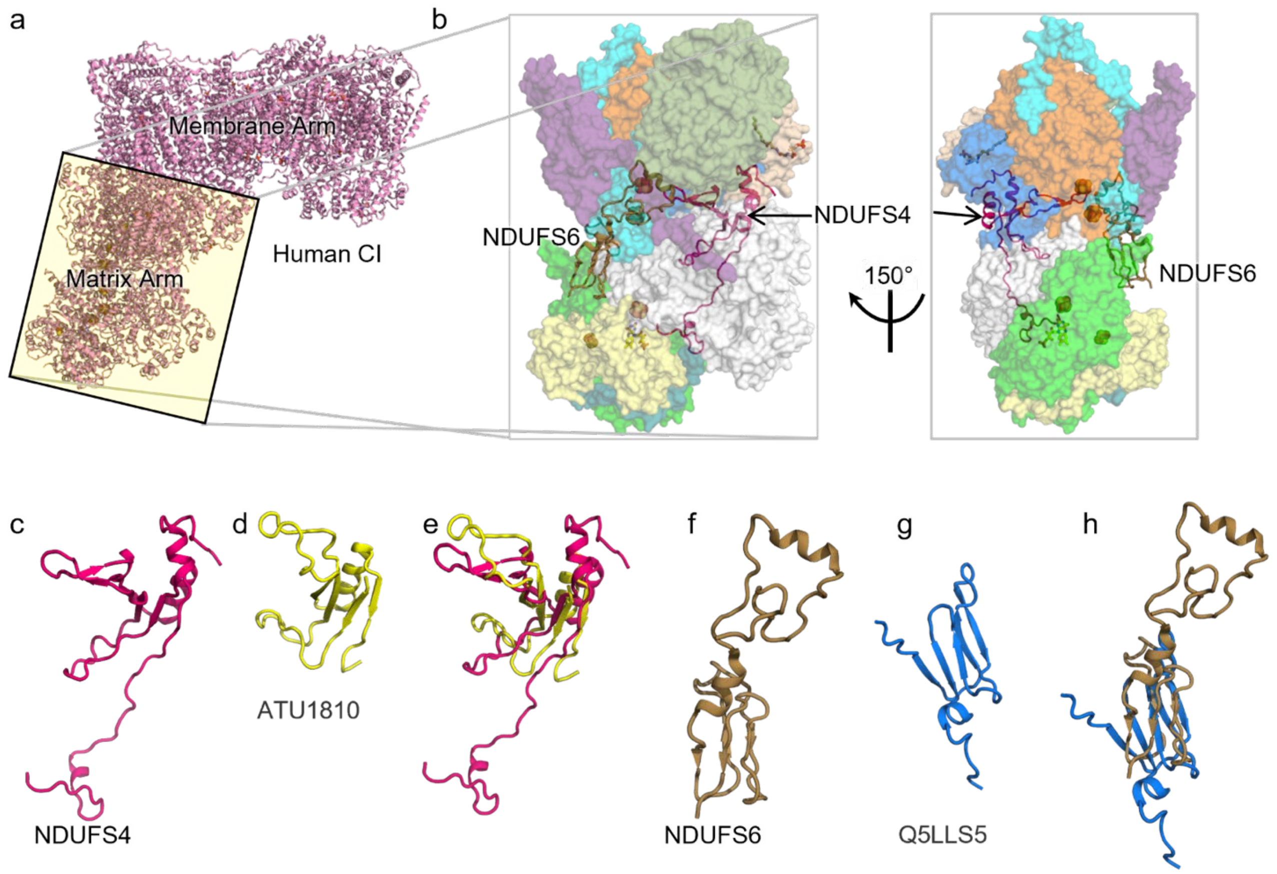

Supplementary Figure 14. Disorder and flexibility in the accessory subunits of mitochondrial complex I.

(a) The cryoEM structure of human respiratory Complex I (pink ribbon, PDB ID 5XTD)[20]. (b) Accessory subunits are found in the matrix arm, which is the soluble region (PDB ID 5XTB)[20]. Two of these accessory subunits provide examples of disorder and dynamics in the assembly of the complex. These are NADH dehydrogenase [ubiquinone] iron-sulfur protein 4 (NDUFS4, magenta ribbon) and NADH dehydrogenase [ubiquinone] iron-sulfur protein 6 (NDUFS6, brown ribbon). (c) In the context of assembled Complex I, NDUFS4 (*hot pink*) is ordered but lacks secondary structure in >60% of the protein. (d) An NMR structure of a homolog from *Agrobacterium tumefaciens* (yellow ribbon, PDB ID 2JYA)[21], which shares 55% sequence similarity and 37% identity with residues 20-111 of NDUFS4. (e) NDUSF4 and the *A. tumefaciens* homolog fail to satisfyingly superpose. The RMS deviation of Cα atoms is > 5Å. The unstructured C-terminus of NDUSF4 is not observed in the structure of the bacterial homolog. (f) The structure of NDUFS6 resected from mammalian Complex I is ordered but lacks secondary structure in >70% of the protein. (g) The NDUFS6 homolog from *Silicibacter pomeroyi* (PDB ID 2JRR)[22] shares 67% sequence similarity and 43% identity with residues 80-119 of NDUFS6. (h) The NMR structure of the *S. pomeroyi* protein fails to superpose well with NDUFS6, with an RMS deviation of Cα atom of 4.8 Å.

Supplementary Tables 1 – 4

Supplementary Table 1. Crystallographic data collection and refinement statistics. Numbers in parentheses indicate values for the highest resolution shell of data.

	SDHA-SDHAF2-SDHAF4	SDHA-SDHAF4
**Data collection**		
Space group	$P2_12_12_1$	$P2_12_12$
Cell dimensions		
a, b, c (Å)	66.0, 103.2,126.2	139.0, 69.3, 72.6
α, ϕ3, ψ (°)	90, 90, 90	90, 90, 90
Resolution (Å)	50-1.52 (1.56-1.52)*	50-1.45 (1.51-1.45)*
R_{sym}	0.081 (0.970)*	0.096 (1.421)*
R_{pim}	0.041 (0.491)*	0.041 (0.614)*
$I/\sigma I$	24.5 (1.7)*	35.6 (1.5)*
Completeness (%)	91.7 (89.0)*	99.8 (100)*
Redundancy	4.2 (4.1)*	7.0 (6.2)*
**Refinement**		
Resolution (Å)	35.5-1.52	28.5-1.45
No. reflections	122165	125470
R_{work}/R_{free}	0.157/0.184	0.154/0.171
No. atoms		
Protein	6083	5185
Ligand	212	146
Water	518	423
B-factors (mean)		
Protein	23.09	26.44
Ligand	31.03	30.38
Water	32.63	36.12
RMS deviations		
bond lengths (Å)	0.008	0.008
bond angles (°)	1.02	0.972

A single crystal was used for each structure. Values in parentheses are for highest-resolution shell.

Supplementary Table 2. Inter-protein interactions in the SDHA-AF2-AF4 assembly intermediate.

S. No.	SDHA	Length	SDHAF2
1	K92 (O)	2.73	Y72(O_{η})
2	T96(O_{γ}^{1})	2.82	S113(O)
3	R97(N_{η}^{1})	2.89	P112(O)
4	R97(N_{η}^{2})	2.87	P112(O)
5	H99(N_{δ}^{1})	3.36	G78(O)
6	V101(O)	2.93	W116(N_{ε}^{1})
7	Q104(N_{ε}^{2})	3.21	Y120(O_{η})
8	R195(N_{η}^{1})	3.19	D46(O_{δ}^{2})
9	R195(N_{η}^{2})	2.41	D46(O_{δ}^{1})
10	H198(N_{ε}^{2})	2.77	S113(O_{γ})
11	S199(O_{γ})	2.77	D46(O_{δ}^{2})
12	H202(N_{δ}^{1})	3.13	S113(O_{γ})
13	H202(N_{ε}^{2})	2.73	I50(O)
14	R210(N_{η}^{2})	3.31	P51(O)
15	E240(O_{ε}^{2})	2.88	R58(N_{η}^{1})
16	E240(O_{ε}^{2})	2.64	R58(N_{η}^{2})
17	H270(O)	2.97	K76(NZ)
18	M388(O)	2.93	N146(N_{δ}^{2})
19	F390(O)	2.78	R152(N)
20	E398(O_{ε}^{1})	3.11	K149(N_{ζ})
21	R507(N_{η}^{1})	2.86	E163(O_{ε}^{1})
22	S509(O_{γ})	2.68	E159(O_{ε}^{2})
23	R512(N_{η}^{2})	3.04	D157(O_{δ}^{1})
24	E564(O_{ε}^{1})	2.92	R75(N_{η}^{2})
25	E564(O_{ε}^{2})	2.79	R75(N_{ε})
26	E564(O_{ε}^{2})	2.79	K76(N_{ζ})
27	E567(O_{ε}^{1})	2.70	K76(N_{ζ})
			**SDHAF4**
1	Q104(O_{ε}^{1})	2.98	R104(N_{η}^{1})
2	Q104(O_{ε}^{1})	3.30	R104(N_{η}^{2})
3	S161(O_{γ})	2.73	E84(O_{ε}^{2})
4	R162(N)	2.92	K85(O)
5	R162(O)	2.83	K85(N)
6	R171(N_{ε})	2.76	E92(O_{ε}^{2})
7	R171(N_{η}^{1})	2.82	E84(O_{ε}^{2})
8	R171(N_{η}^{2})	2.92	E84(O_{ε}^{1})
9	R171(N_{η}^{2})	2.87	E92(O_{ε}^{1})
10	A193(N)	3.13	G86(O)
11	D194(O_{δ}^{1})	2.79	R101(N_{η}^{1})
12	D194(O_{δ}^{2})	3.03	R89(N)
13	L306(O)	2.79	C105(N)
14	T308(N)	2.88	G103(O)
15	T308(O_{γ}^{1})	2.65	D98(O_{δ}^{2})
16	R379(O)	2.84	R95(N_{η}^{2})
17	A454(N)	2.99	D107(O_{δ}^{2})
18	L306(O)	2.79	C105(N)
19	T308(N)	2.88	G103(O)
20	T308(O_{γ}^{1})	2.65	D98(O_{δ}^{2})
21	R379(O)	2.84	R95(N_{η}^{2})
22	T386(O_{γ}^{1})	2.93	K102(O)

Supplementary Table 3: Acquisition conditions for the NMR experiments reported.

**Experiment**	**Complex acquisition matrix [pts]**	**Sweep width 2D: F2 x F1 [Hz] 3D: F3 x F2 x F1 [Hz]**	**Offsets F1, F2, F3 [ppm]**	**Number of scans**	**NUS amount**	**Expt time [hrs]**
hacacoNcaNCO	1k x 64 x 48	4529 x 1825 x 1824	173.0, 117.25, 117.25	NS=48	traditional	55.6
hacaCOncaNCO	1k x 64 x 48	4529 x 1825 x 1245	173.0, 117.25, 173.0	NS=48	traditional	55.5
CON	1k x 256	4529 x 4106	173.0, 117.25	NS=8	traditional	0.8
HSQC	2k x 128	10822 x 2555	173.0, 119.0	NS=2	traditional	0.1
HNCO	2k x 48 x 96	10822 x 1825 x 1245	4.70, 117.25, 173.0	NS=8	25%	3.1
HNcaCO	2k x 48 x 64	10822 x 2007 x 1245	4.70, 117.25, 173.0	NS=16	40%	6.6
HNCA	2k x 48 x 128	10822 x 1825 x 6792	4.70, 117.25, 57.0	NS=8	25%	4.1
HNcoCA	2k x 48 x 96	10822 x 2007 x 8150	4.70, 117.25, 52.0	NS=8	40%	5.0
CBCAcoNH	2k x 48 x 128	10822 x 1825 x 18111	4.70, 117.25, 45.0	NS=8	traditional	17.0
HNCACB	2k x 64 x 168	10822 x 2007 x 13583	4.70, 117.25, 42.0	NS=48	40%	69.5

Supplementary Table 4. Inter-protein interactions in the SDHA-AF4 assembly intermediate.

**S. No.**	**SDHA**	**Length**	**SDHAF4**
1	Q104(O_{ε}^{1})	2.82	R104(N_{η}^{2})
2	S161(O_{γ})	2.61	E84(O_{ε}^{2})
3	R162(N)	2.95	K85(O)
4	R162(O)	2.84	K85(N)
5	R171(N_{ε})	2.82	E92(O_{ε}^{2})
6	R171(N_{η}^{1})	2.86	E84(O_{ε}2)
7	R171(N_{η}^{2})	2.89	E84(O_{ε}^{1})
8	R171(N_{η}^{2})	2.89	E92(O_{ε}^{1})
9	A193(N)	3.10	G86(O)
10	D194(O_{δ}^{1})	2.81	R101(N_{η}^{1})
11	D194(O_{δ}^{2})	2.93	R89(N)
12	H407(N_{ε}^{2})	3.28	D107(O_{δ}^{1})
13	R451(N_{η}^{1})	2.78	D107(O_{δ}^{1})
14	R451(N_{η}^{2})	3.08	D107(O_{δ}^{1})
15	R451(N_{η}^{2})	3.04	F108(OXT)
16	A454(N)	2.96	D107(O_{δ}^{2})
17	L306(O)	2.83	C105(N)
18	T308(N)	2.88	G103(O)
19	T308(O_{γ}^{1})	2.82	D98(O_{δ}^{1})
20	E309(N)	2.91	D98(O_{δ}^{2})
21	E314(O_{ε}^{2})	2.60	Y96(O_{η})
22	R379(O)	3.12	R95(N_{η}^{2})
23	E385(O_{ε}^{1})	2.85	E69(N)
24	E385(O_{ε}^{1})	2.99	K102(N_{ζ})
25	T386(O_{γ}^{1})	2.68	K102(O)

SUPPLEMENTARY REFERENCES

1 Eletsky, A. *et al.* Solution NMR structure of yeast succinate dehydrogenase flavinylation factor Sdh5 reveals a putative Sdh1 binding site. *Biochemistry* **51**, 8475-8477, doi:10.1021/bi301171u (2012).

2 Sharma, P., Maklashina, E., Cecchini, G. & Iverson, T. M. The roles of SDHAF2 and dicarboxylate in covalent flavinylation of SDHA, the human complex II flavoprotein. *Proc Natl Acad Sci U S A* **117**, 23548-23556, doi:10.1073/pnas.2007391117 (2020).

3 Borcherds, W. M. & Daughdrill, G. W. Using NMR Chemical Shifts to Determine Residue-Specific Secondary Structure Populations for Intrinsically Disordered Proteins. *Methods Enzymol* **611**, 101-136, doi:10.1016/bs.mie.2018.09.011 (2018).

4 Berjanskii, M. V. & Wishart, D. S. A simple method to predict protein flexibility using secondary chemical shifts. *J Am Chem Soc* **127**, 14970-14971, doi:10.1021/ja054842f (2005).

5 Nielsen, J. T. & Mulder, F. A. A. CheSPI: chemical shift secondary structure population inference. *J Biomol NMR* **75**, 273-291, doi:10.1007/s10858-021-00374-w (2021).

6 Emsley, P. & Cowtan, K. Coot: model-building tools for molecular graphics. *Acta Crystallogr D Biol Crystallogr* **60**, 2126-2132, doi:10.1107/S0907444904019158 (2004).

7 Kudryavtseva, A. V. *et al.* Mutation profiling in eight cases of vagal paragangliomas. *BMC Med Genomics* **13**, 115, doi:10.1186/s12920-020-00763-4 (2020).

8 Sun, F. *et al.* Crystal structure of mitochondrial respiratory membrane protein complex II. *Cell* **121**, 1043-1057, doi:10.1016/j.cell.2005.05.025 (2005).

9 Furst, J. *et al.* ICln159 folds into a pleckstrin homology domain-like structure. Interaction with kinases and the splicing factor LSm4. *J Biol Chem* **280**, 31276-31282, doi:10.1074/jbc.M500541200 (2005).

10 Grimm, C. *et al.* Structural basis of assembly chaperone- mediated snRNP formation. *Mol Cell* **49**, 692-703, doi:10.1016/j.molcel.2012.12.009 (2013).

11 Abajian, C., Yatsunyk, L. A., Ramirez, B. E. & Rosenzweig, A. C. Yeast cox17 solution structure and Copper(I) binding. *J Biol Chem* **279**, 53584-53592, doi:10.1074/jbc.M408099200 (2004).

12 Banci, L. *et al.* A hint for the function of human Sco1 from different structures. *Proc Natl Acad Sci U S A* **103**, 8595-8600, doi:10.1073/pnas.0601375103 (2006).

13 Zhan, X., Gimenez, L. E., Gurevich, V. V. & Spiller, B. W. Crystal structure of arrestin-3 reveals the basis of the difference in receptor binding between two non-visual subtypes. *J Mol Biol* **406**, 467-478, doi:10.1016/j.jmb.2010.12.034 (2011).

14 Chen, Q. *et al.* Structural basis of arrestin-3 activation and signaling. *Nat Commun* **8**, 1427, doi:10.1038/s41467-017-01218-8 (2017).

15 Huang, W. *et al.* Structure of the neurotensin receptor 1 in complex with beta-arrestin 1. *Nature* **579**, 303-308, doi:10.1038/s41586-020-1953-1 (2020).

16 Staus, D. P. *et al.* Structure of the M2 muscarinic receptor-beta-arrestin complex in a lipid nanodisc. *Nature* **579**, 297-302, doi:10.1038/s41586-020-1954-0 (2020).

17 Laughlin, T. G. *et al.* Architecture and self-assembly of the jumbo bacteriophage nuclear shell. *Nature* **608**, 429-435, doi:10.1038/s41586-022-05013-4 (2022).

18 Chaikeeratisak, V. *et al.* Assembly of a nucleus-like structure during viral replication in bacteria. *Science* **355**, 194-197, doi:10.1126/science.aal2130 (2017).

19 Mendoza, S. D. *et al.* A bacteriophage nucleus-like compartment shields DNA from CRISPR nucleases. *Nature* **577**, 244-248, doi:10.1038/s41586-019-1786-y (2020).

20 Guo, R., Zong, S., Wu, M., Gu, J. & Yang, M. Architecture of Human Mitochondrial Respiratory Megacomplex I2III2IV2. *Cell* **170**, 1247-1257 e1212, doi:10.1016/j.cell.2017.07.050 (2017).

21 Lemak, A. *et al.* NMR solution structure of protein ATU1810 from Agrobacterium tumefaciens. Northeast Structural Genomics Consortium target AtR23, Ontario Centre for Structural Proteomics Target ATC1776. *Northeast Structural Genomics Consortium (NESG), Ontario Centre for Structural Proteomics (OCSP)*, doi:10.2210/pdb2JYA/pdb (2007).

22 Swapna, G. V. T. *et al.* Solution NMR Structure of Q5LLS5 from Silicibacter pomeroyi. Northeast Structural Genomics Consortium target SiR90. *Northeast Structural Genomics Consortium (NESG)*, doi:10.2210/pdb2JRR/pdb (2007).
