# Supplementary material for: Disordered-to-ordered transitions in assembly factors allow the complex II catalytic subunit to switch binding partners
Source: Nat Commun. 2024 Jan 11;15:473. doi: 10.1038/s41467-023-44563-7 (PMC10784507; doi:10.1038/s41467-023-44563-7)
Supplement: Supplementary file 3 — Reporting Summary [file 41467_2023_44563_MOESM3_ESM.pdf]

nature portfolio

Corresponding author(s): Tina Iverson

Last updated by author(s): Dec 6, 2023

Reporting Summary

Nature Portfolio wishes to improve the reproducibility of the work that we publish. This form provides structure for consistency and transparency in reporting. For further information on Nature Portfolio policies, see our Editorial Policies and the Editorial Policy Checklist.

Statistics

For all statistical analyses, confirm that the following items are present in the figure legend, table legend, main text, or Methods section.

n/a	Confirmed	
☐	☒	The exact sample size (*n*) for each experimental group/condition, given as a discrete number and unit of measurement
☐	☒	A statement on whether measurements were taken from distinct samples or whether the same sample was measured repeatedly
☐	☒	The statistical test(s) used AND whether they are one- or two-sided<br>*Only common tests should be described solely by name; describe more complex techniques in the Methods section.*
☒	☐	A description of all covariates tested
☒	☐	A description of any assumptions or corrections, such as tests of normality and adjustment for multiple comparisons
☒	☐	A full description of the statistical parameters including central tendency (e.g. means) or other basic estimates (e.g. regression coefficient) AND variation (e.g. standard deviation) or associated estimates of uncertainty (e.g. confidence intervals)
☐	☒	For null hypothesis testing, the test statistic (e.g. *F*, *t*, *r*) with confidence intervals, effect sizes, degrees of freedom and *P* value noted<br>*Give P values as exact values whenever suitable.*
☒	☐	For Bayesian analysis, information on the choice of priors and Markov chain Monte Carlo settings
☒	☐	For hierarchical and complex designs, identification of the appropriate level for tests and full reporting of outcomes
☒	☐	Estimates of effect sizes (e.g. Cohen's *d*, Pearson's *r*), indicating how they were calculated

Our web collection on statistics for biologists contains articles on many of the points above.

Software and code

Policy information about availability of computer code

Data collection	For crystal structure determination, data for both SDHA-AF2-AF4 and SDHA-AF4 crystals was collected at APS Beamline 21-ID-F.
Data analysis	ImageJ 1.53k; Java 1.8.0_172 (64 bit); Excel - Microsoft 365 Apps For Business Version 2208 Build 16.0.15601.20072, 64-bit); Graphpad Prism 8.4.3 (686) was used for image analysis and biochemical studies.<br>For crystal structure analysis HKL-2000 version 715.5 was used for data reduction and scaling. Phenix version 1.19.2_4158, was used for molecular replacement and coot version 0.9.6 was used for refinement.

For manuscripts utilizing custom algorithms or software that are central to the research but not yet described in published literature, software must be made available to editors and reviewers. We strongly encourage code deposition in a community repository (e.g. GitHub). See the Nature Portfolio guidelines for submitting code & software for further information.

Data

Policy information about availability of data

All manuscripts must include a data availability statement. This statement should provide the following information, where applicable:

- Accession codes, unique identifiers, or web links for publicly available datasets
- A description of any restrictions on data availability
- For clinical datasets or third party data, please ensure that the statement adheres to our policy

PDB coordinates and processed diffraction data generated in this study have been deposited in the Protein Data Bank (https://wwpdb.org/) with accession codes

8DYD (https://doi.org/10.2210/pdb8DYD/pdb) and 8DYE (https://doi.org/10.2210/pdb8DYE/pdb). Raw diffraction data have been deposited with SBGrid (http://data.sbgrid.org) with accession codes and direct hyperlinks of 954 (doi:10.15785/SBGRID/954) and 955 (doi:10.15785/SBGRID/955). NMR Chemical shift data for SDHAF4 have been deposited in the BMRB databank (http://www.bmrb.wisc.edu), retrievable under the accession number 52207 (doi: 10.13018/BMR52207). Source data are provided with this paper.

Other PDB entries used in this study and their description:
The human SDHA-AF2 complex, PDB entry 3VAX [https://doi.org/10.2210/pdb3VAX/pdb] was used for molecular replacement. Porcine mitochondrial Complex II, PDB entry 3SFD [https://doi.org/10.2210/pdb3SFD/pdb] was used for showing assembled complex II in Figure 7.
The following PDBs are cited in the discussion: 4F7U [https://doi.org/10.2210/pdb4F7U/pdb], 1ZYI [https://doi.org/10.2210/pdb1ZYI/pdb], 1U96 [https://doi.org/10.2210/pdb1U96/pdb], 1U97 [https://doi.org/10.2210/pdb1U97/pdb], 2GT5 [https://doi.org/10.2210/pdb2GT5/pdb], 2GQK [https://doi.org/10.2210/pdb2GQK/pdb], 2GQM[https://doi.org/10.2210/pdb2GQM/pdb], 3P2D [https://doi.org/10.2210/pdb3P2D/pdb], 5TV1 [https://doi.org/10.2210/pdb5TV1/pdb], 7SQU [https://doi.org/10.2210/pdb7SQU/pdb], 5XTD [https://doi.org/10.2210/pdb5XTD/pdb], 2JYA [https://doi.org/10.2210/pdb2JYA/pdb], 2JRR [https://doi.org/10.2210/pdb2JRR/pdb].

Human research participants

Policy information about studies involving human research participants and Sex and Gender in Research.

Reporting on sex and gender	n/a
Population characteristics	n/a
Recruitment	n/a
Ethics oversight	n/a

Note that full information on the approval of the study protocol must also be provided in the manuscript.

Field-specific reporting

Please select the one below that is the best fit for your research. If you are not sure, read the appropriate sections before making your selection.

☒ Life sciences ☐ Behavioural & social sciences ☐ Ecological, evolutionary & environmental sciences

For a reference copy of the document with all sections, see nature.com/documents/nr-reporting-summary-flat.pdf

Life sciences study design

All studies must disclose on these points even when the disclosure is negative.

Sample size	The number of cells cannot be exactly determined for cell line studies (only one cell line was constantly used per group), but was normalized to GAPDH quantity. The n in the figures and text represent number of independent experiments
Data exclusions	For experiments involving hPheo1 cell lines, data were excluded e.g. in the case of WB, when the loading control was not consistent for all groups.<br>During crystal structure analysis, the diffraction data which was beyond the preferred range is excluded from further analysis.
Replication	The number of replicates was selected in order to minimize the intra-group variance. The cell-line experiments are illustration of 3 independent IP replications using 3 independent biological samples. For the in vitro protein studies 3-4 replications is a common approach in the field. All the attempts to to replicate the experiments were successful.
Randomization	Randomization was not performed. However, the groups were treated independently.
Blinding	Blinding was not performed.

Reporting for specific materials, systems and methods

We require information from authors about some types of materials, experimental systems and methods used in many studies. Here, indicate whether each material, system or method listed is relevant to your study. If you are not sure if a list item applies to your research, read the appropriate section before selecting a response.

Materials & experimental systems

n/a	Involved in the study
☐	☒ Antibodies
☐	☒ Eukaryotic cell lines
☒	☐ Palaeontology and archaeology
☒	☐ Animals and other organisms
☒	☐ Clinical data
☒	☐ Dual use research of concern

Methods

n/a	Involved in the study
☒	☐ ChIP-seq
☒	☐ Flow cytometry
☒	☐ MRI-based neuroimaging

Antibodies

Antibodies used

The following primary antibodies were used in this study : anti-SDHA (ab14715, Abcam), anti-SDHA (Cell Signaling 11998), anti-SDHB (ab14714, Abcam), anti-SDHAF2 (45849, Cell Signaling Technology), anti-SDHAF4 (NBP1-86324, Novus), anti-FLAG (GenScript A00187) and for loading controls anti-Hsp60 (12165, Cell Signaling Technology), anti-VDAC1 (ab15895; Abcam) or anti-GAPDH (5174; Cell Signaling Technology). Secondary antibodies used are goat anti-rabbit (170-6515, Biorad) or goat anti-mouse (170-6516, Biorad).

Validation

All antibodies were validated by the manufacturers.

Eukaryotic cell lines

Policy information about cell lines and Sex and Gender in Research

Cell line source(s)

Source of hPheo1 cell line: Hans Ghayee (co-author) as reported in Ghayee HK, Bhagwandin VJ, Stastny V, Click A, Ding LH, Mizrachi D, Zou YS, Chari R, Lam WL, Bachoo RM, Smith AL, Story MD, Sidhu S, Robinson BG, Nwariaku FE, Gazdar AF, Auchus RJ, Shay JW. Progenitor cell line (hPheo1) derived from a human pheochromocytoma tumor. PLoS One. 2013 Jun 13;8(6):e65624. doi: 10.1371/journal.pone.0065624.
Sex; Female

Authentication

The hPheo1 cell line was not specifically validated.

Mycoplasma contamination

Cell lines were tested negative for mycoplasma contamination.

Commonly misidentified lines
(See ICLAC register)

none
